# Genetic evidence for causal associations between common cell-derived signaling molecules and sleep disorders and the mediating role of metabolites

**DOI:** 10.1097/MD.0000000000044301

**Published:** 2025-09-05

**Authors:** Liqun Li, Xiaomei Tang, Jinjing Tan, Jing Yan, Chunmei Wang, Jiaqi Li, Zhiwen Shen, Sheng Xie

**Affiliations:** a Graduate School of Guangxi University of Chinese Medicine, Nanning, Guangxi, China; b The First Affiliated Hospital of Guangxi University of Chinese Medicine, Nanning, Guangxi, China.

**Keywords:** cell-derived signaling molecules, mediation analysis, Mendelian randomization, sleep disorder

## Abstract

Although the potential causal associations between cell-derived signaling molecules and sleep disorder (SD) have been reported, contradictions remain. This study assessed the causal effects and the mediating role of 1400 metabolites among 91 cell-derived signaling molecules and SD from a genetic perspective by performing Mendelian randomization (MR) analyses. Genetic instruments derived from publicly available genome-wide association studies were employed in this study, including 49,880 SD cases and 358,194 controls. Summary statistics of 1400 circulating metabolites were obtained from a cohort of 8299 individuals. The 91 cell-derived signaling molecules were derived from genome-wide association studies data from 11 cohorts comprising 14,824 samples. Multiple statistical analyses were introduced in this study, with inverse variance weighted as the core analysis method, supplemented by 4 additional methods. Besides, various sensitivity analyses were employed to identify horizontal pleiotropy and heterogeneity, thereby evaluating the robustness of the results. Forward MR analysis indicated positive associations between SD and the levels of eotaxin (OR = 1.041, 95% CI: 1.001–1.084, *P* = .046), CUB domain-containing protein 1 (OR = 1.042, 95% CI: 1.008–1.077, *P* = .016), interleukin-20 receptor subunit alpha (IL-20RA) (OR = 1.086, 95% CI: 1.031–1.143, *P* = .002), while the levels of CD40L receptor (OR = 0.968, 95% CI: 0.942–0.994, *P* = .018), glial cell line-derived neurotrophic factor (OR = 0.947, 95% CI: 0.910–0.986, *P* = .009) act as the opposite. Reverse MR analysis pointed out that the genetic susceptibility to SD raised interleukin-5 levels. According to the mediation analysis, N-lactoyl-tyrosine levels mediated the increased risks of SD associated with elevated IL-20RA levels, with a mediation effect of 0.009 (95% CI: 0.001–0.018, *P* = .034), accounting for 11.5% of the total. The study proved the causal associations between 91 cell-derived signaling molecules and SD, confirming that eotaxin, CUB domain-containing protein 1, and IL-20RA may increase the risk of SD, while CD40L receptor and glial cell line-derived neurotrophic factor may act as the opposite. Besides, the study provided abundant evidence for the potential mediating effect of N-lactoyl-tyrosine in the pathway linking IL-20RA and SD risk. To summarize, the findings of this study may benefit the understanding of the pathogenic mechanisms through which cell-derived signaling molecules influence SD.

## 1. Introduction

Sleep disorders (SD) refer to a comprehensive concept encompassing disorders that initiate and maintain normal sleep states, disorders manifested as excessive sleep tendencies, disorders with disrupted sleep wake rhythms, and functional disorders associated with sleep processes, specific sleep stages, or partially awakened states during sleep.^[1]^ The prevalence of SD widely ranged from 5% to 50%, depending on the definition used. To exemplify, the prevalence reaches 30% to 36% when defined as the presence of at least 1 symptom of insomnia; while it drops to 10% to 25% when defined by dissatisfaction with sleep quality or quantity. And when defined by impaired daytime functioning, the prevalence decreases to 10% to 15%.^[2]^ Approximately one-third of the adults worldwide suffer from insomnia symptoms, placing a significant burden on both global health and the economy. Currently, chemical drugs used to treat insomnia include benzodiazepine receptor agonists, melatonin, and its receptor agonists, antidepressants, antipsychotics, and antihistamines. While these drugs have shown effectiveness in treating acute insomnia, their safety, tolerability, and variable clinical efficacy limit the further application in managing chronic insomnia.^[3–5]^ Furthermore, a report indicated higher morbidities of chronic pain, hypertension, gastrointestinal issues, repository disorders, heart disease, urinary problems, and neurological diseases among patients with chronic insomnia.^[6]^ These patients were also haunted by a 55% increased risk of stroke and a 28% higher risk of coronary heart disease, as well as rising mortality of cardiovascular diseases by 33%.^[7]^ The evidence underscore the significant risk that SD may pose to multiple organ systems, severely threatening patients’ physical and psychological health. Therefore, there is an urgent need to enhance our understanding of the SD pathogenesis, improve SD treatment protocols and preventive strategies to effectively manage SD.

Multiple studies have revealed the complex interactions between SD and cell-derived signaling molecules. However, the exact causal associations in this process remain unclear. A clinical study showed that serum levels of inflammatory mediators, including serum amyloid protein A, tumor necrosis factor (TNF-α), and granulocyte-macrophage colony-stimulating factor witness significant increase in SD patients. In contrast, the RANTES levels showed the opposite. Additionally, these variations demonstrated certain associations with the severity of insomnia to some extent.^[8]^ Another study has reported that the increased sleep duration may correspond with the rised levels of C-reactive protein and interleukin-6 (IL-6), while reduced sleep duration is related to increased TNFα levels.^[9]^ A clinical study has found that interleukin-20 receptor subunit alpha (IL-20RA) plays a crucial role in fatty acid metabolism, oxygen binding and transportation, and hormone activity.^[10]^ SD may cause substantial changes in metabolites, for example, lipoproteins, triglycerides, isoleucine, valine, choline, and phosphocholine, with all fatty acid components decreasing, which indicated post-SD deficiency in fatty-acetal metabolism.^[11]^ Another study has suggested that the consumption of fatty acids containing 6 to 10 carbon atoms may benefit sleep, potentially by providing nutrients or activating sensory neurons.^[12]^ However, the evidence above are all from observational studies, which susceptible to unanticipated confounding factors or reverse causality. Thus, the debate over whether the cell-derived signaling molecules act as the cause or consequence of the progression of SD remains unresolved. In light of this, a reliable causal inferring methodology becomes necessary to clarify the causal associations between cell-derived signaling molecules and SD, thereby providing scientific insights for treating SD.

As an epidemiological method, Mendelian randomization (MR) employs GWAS to summarize genetic variation in data and infer causal associations between exposures and outcomes.^[13]^ Since alleles are randomly assigned during meiosis, MR can minimize the impact induced by confounding factors and reverse causality, thereby providing solid evidence for causal inferences.^[14,15]^ Although widely applied in explorations of disease risks, MR has not been involved in studies investigating the causal association between cell-derived signaling molecules and SD or the mediating roles circulating metabolites may play in this process. Based on the aforementioned background, we raised the scientific question of whether genetic-predicted cell-derived signaling molecules play key roles in the onset and progression of SD. If so, do blood metabolites causally bridge the cell-derived signaling molecules and SD? To provide possible answers to these questions, we applied open GWAS data in this study to perform a 2-sample bidirectional MR analysis to investigate latent causal associations between cell-derived signaling molecules and SD. A 2-step MR was introduced to explore the mediating effect of circulating metabolites in this process.

## 2. Materials and methods

### 2.1. Study design

**Figure 1.**
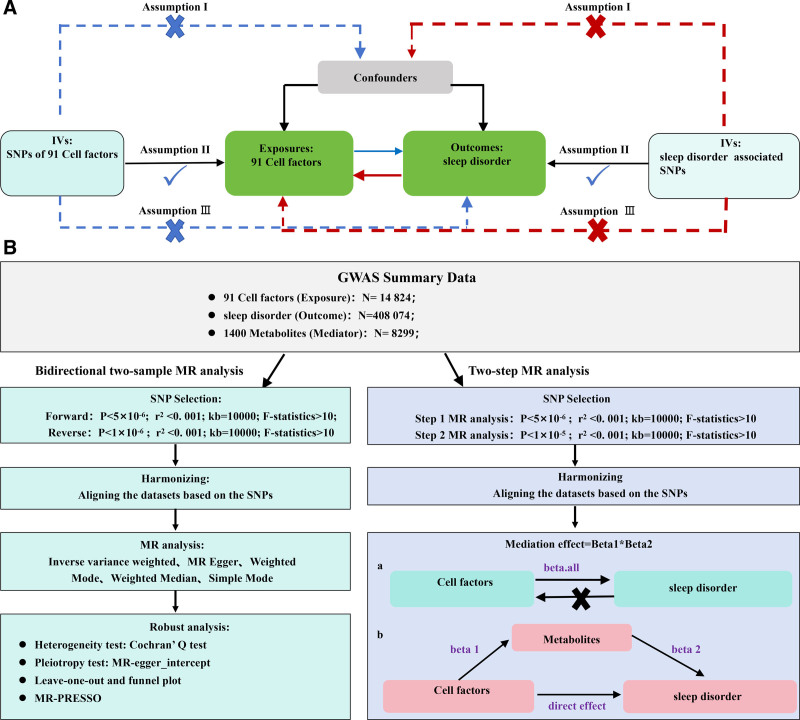
(A) Schematic diagram of MR principles. (B) Flowchart of the bidirectional 2-sample MR analysis and mediation analysis. MR = Mendelian randomization.

This study utilized GWAS summary data for 91 cell-derived signaling molecules and SD. Multiple single nucleotide polymorphisms (SNPs) were selected to represent genetic variants and were used as the instrumental variables (IVs) in a 2-sample MR analysis, which was conducted to assess the causal associations between 91 cell-derived signaling molecules and SD, with a 2-step MR analysis for investigating mediating effects of 1400 metabolites. The study is rooted in 3 fundamental assumptions of MR: relevance: the genetic variants introduced as IVs must be highly relevant to the exposure. Independence: the selected genetic variants must be independent of confounding factors, with no pleiotropic association. Exclusiveness: the exposure should be the sole approach that the genetic variants may influence the outcome. No extra ethical approval is required since this study was based on the summary genetic data from GWAS. The study was designed and drafted according to STROBE-MR, the reporting guidelines of MR studies.^[16]^ The study design is demonstrated in Figure 1.

### 2.2. Data sources

GWAS data involved in this study is available at (https://www.phpc.cam.ac.uk/ceu/proteins) in full version, including data on 91 cell-derived signaling molecules and 14,824 samples.^[17]^ Details for data management can be derived from the original publication. GWAS summary data of SD can be downloaded via (https://www.finngen.fi/en), with ID “finngen_R10_SLEEP.gz,” involving 49,880 SD cases and 358,194 controls, totaling 408,029 participants. GWAS data of 1400 blood metabolites was sourced from NHGRI-EBI GWAS Catalog (https://www.ebi.ac.uk/gwas/), containing 8299 samples.^[18]^ The accession numbers of European GWAS data are CST90199621 to 902010209. The study populations were all European, avoiding bias due to racial differences.

### 2.3. Selection of genetic IVs

We set a genome-wide significance threshold of *P* < 1e-05 to screen SNPs when the cell-derived signaling molecules, SD, and metabolites were selected as exposures, to ensure the strong relevance between selected SNPs and the exposures. Second, to guarantee the independence of these SNPs, we set the parameters to clump_kb = 10,000 and clump_*r*^2^ = 0.001 to filter out SNPs with linkage disequilibrium. Finally, *F*-statistics (*F* > 10) was introduced to evaluate the strength of each SNP to ensure the reliability of SNPs. The screening criteria secure the independence and exclusiveness of genetic varians involved in this study. The SNP features involved in this study have been summarized in Tables S1 to S3, Supplemental Digital Content, https://links.lww.com/MD/P869.

### 2.4. Bidirectional MR analysis

We performed a bidirectional 2-sample MR analysis to evaluate the causal associations between 91 cell-derived signaling molecules and SD. Inverse variance weighted (IVW), weighted mode, MR-Egger, simple mode, and weighted median were the 5 methods applied, in which the IVW was the major and determining one. Cochran Q test was employed to determine if heterogeneity occurs (*P* < .05) among the SNPs.^[19]^ The MR-Egger intercept test was performed to determine possible pleiotropy (*P* < .05).^[20]^ If pleiotropy detected, MR Pleiotropy Residual Sum and Outlier (MR-PRESSO) would be applied for outlier removal, then to reevaluate and correct for horizontal pleiotropy.^[21]^ A leave-one-out (LOO) test and funnel plots were utilized to determine the robustness of results and identify outliers. All the aforementioned data analyses were performed using R software (version 4.4.0; R Foundation for Statistical Computing, Vienna, Austria) with the Ieugwasr, gwasglue, gwasvcf, ieugwasr, MRInstrumentsR, and other relevant packages.

### 2.5. Mediation analysis

We employed a 2-step MR for the mediation analysis of 1400 blood metabolites to investigate their possible roles as mediators in the causal associations between the cell-derived signaling molecules and SD (Fig. 1). First of all, βall was obtained by MR analysis between cell-derived signaling molecules and SD. Then MR analysis was performed to explore the impact of cell-derived signaling molecules and metabolites, yielding β1. Finally, MR analysis was performed between metabolites and SD to source β2. The formulation (β1 × β2) was finally applied for calculating mediation effects.

## 3. Results

### 3.1. Bidirectional 2-step MR analysis

#### 3.1.1. Causal effect of cell-derived signaling molecules on SD

**Figure 2.**
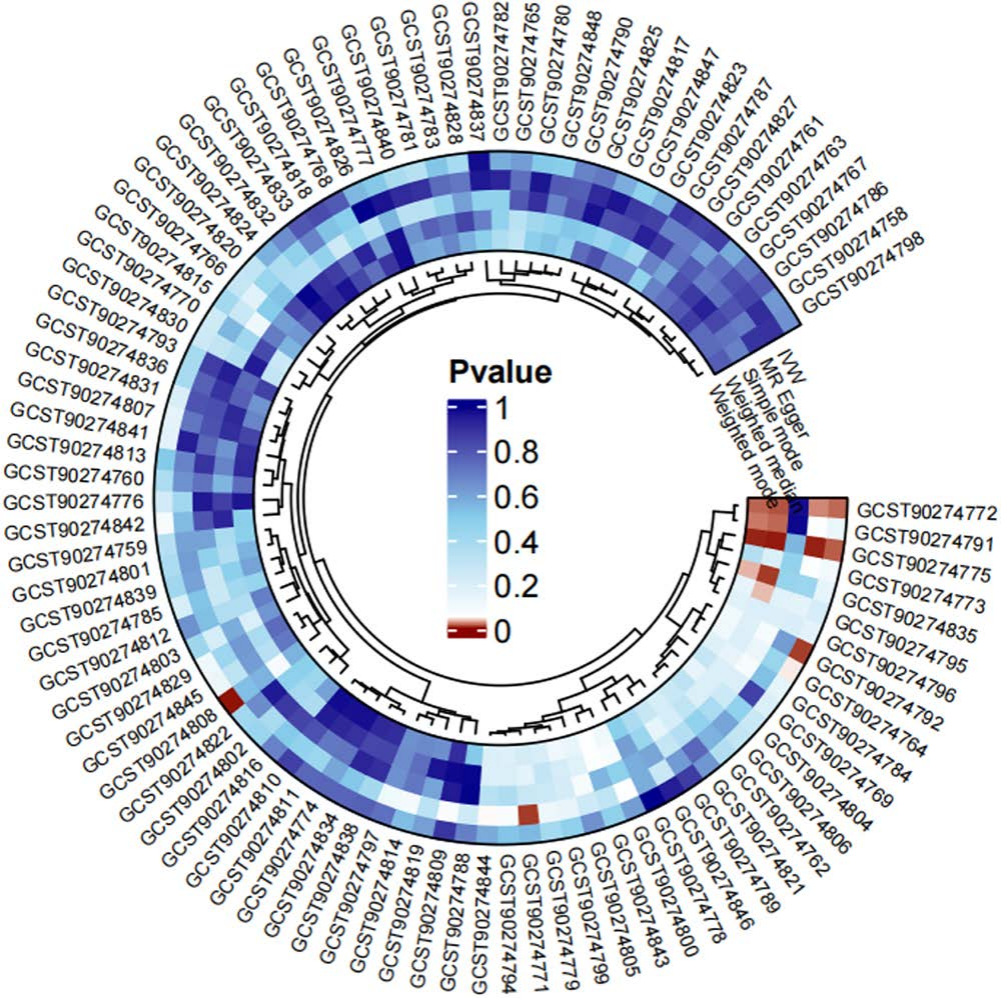
Circle diagram of causal association between 91 cell-derived signaling molecules and SD from the forward MR analysis. MR = Mendelian randomization, SD = sleep disorders.

**Figure 3.**
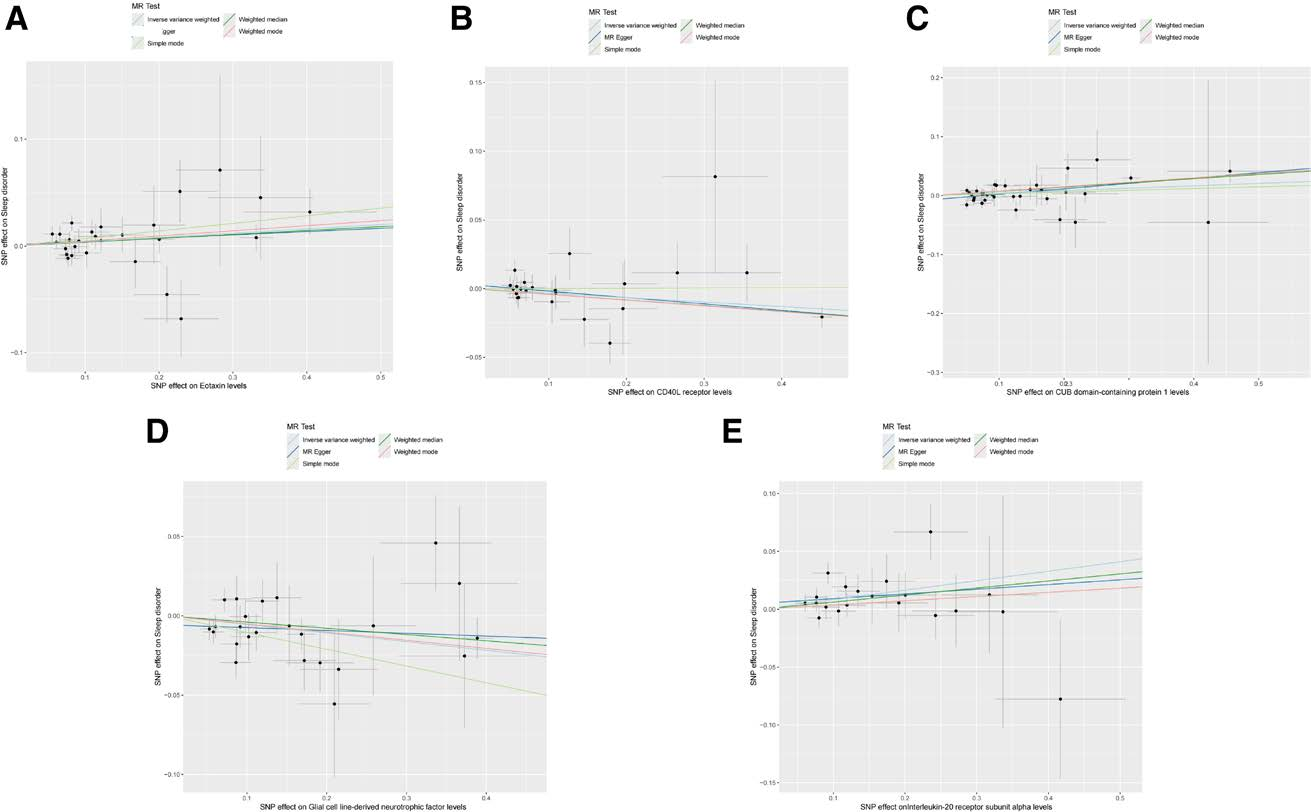
Scatter plot of causal effects between 5 significantly associated cell-derived signaling molecules and SD from the forward MR analysis. (A) Scatter plot of the causal effect between CCL11 and SD. (B) Scatter plot of the causal effect between CD40 and SD. (C) Scatter plot of the causal effect between CDCP1 and SD. (D) Scatter plot of the causal effect between GDNF and SD. (E) Scatter plot of the causal effect between IL-20RA and SD. CD40 = CD40L receptor, CDCP1 = CUB domain-containing protein 1, CCL11 = eotaxin, GDNF = glial cell line-derived neurotrophic factor, MR = Mendelian randomization, SD = sleep disorders.

After the aforementioned filtering steps, all selected SNPs were found robust, with *F*-statistics ranging from 19.51 to 3549.33 (Table S1, Supplemental Digital Content, https://links.lww.com/MD/P869). These SNPs were then utilized for forward MR analysis to evaluate the impact of cell-derived signaling molecules on SD. Results of different MR methods were listed in the Table S4, Supplemental Digital Content, https://links.lww.com/MD/P869. The IVW results revealed that the levels of 5 cell-derived signaling molecules were significantly associated with SD, while other 86 were detected contrary (Fig. 2). Specifically, eotaxin (CCL11) (OR = 1.041, 95% CI: 1.001–1.084, *P* = .046), CUB domain-containing protein 1 (CDCP1) (OR = 1.042, 95% CI: 1.008–1.077, *P* = .016), IL-20RA (OR = 1.086, 95% CI: 1.031–1.143, *P* = .002) may rise the SD risk, while CD40L receptor (CD40) (OR = 0.968, 95% CI: 0.942–0.994, *P* = .018) and glial cell line-derived neurotrophic factor (GDNF) (OR = 0.947, 95% CI: 0.910–0.986, *P* = .009) exhibited protective effects against SD (Fig. 3). According to Cochran Q test, the above 5 inflammatory cytokines performed no heterogeneity. Although the MR-Egger intercept test detected statistical significance in CDCP1, further examination and calibration using MR-PRESSO found no horizontal pleiotropy. The remaining 4 cell-derived signaling molecules did not show statistical significance in the MR-Egger and MR-PRESSO tests, indicating the absence of horizontal pleiotropy (Table S8, Supplemental Digital Content, https://links.lww.com/MD/P869). Besides, the LOO test proved that any single SNP did not influence MR outcomes. To conclude, the sensitivity tests ensured the robustness of our results.

#### 3.1.2. Effect of SD on cell-derived signaling molecules

**Figure 4.**
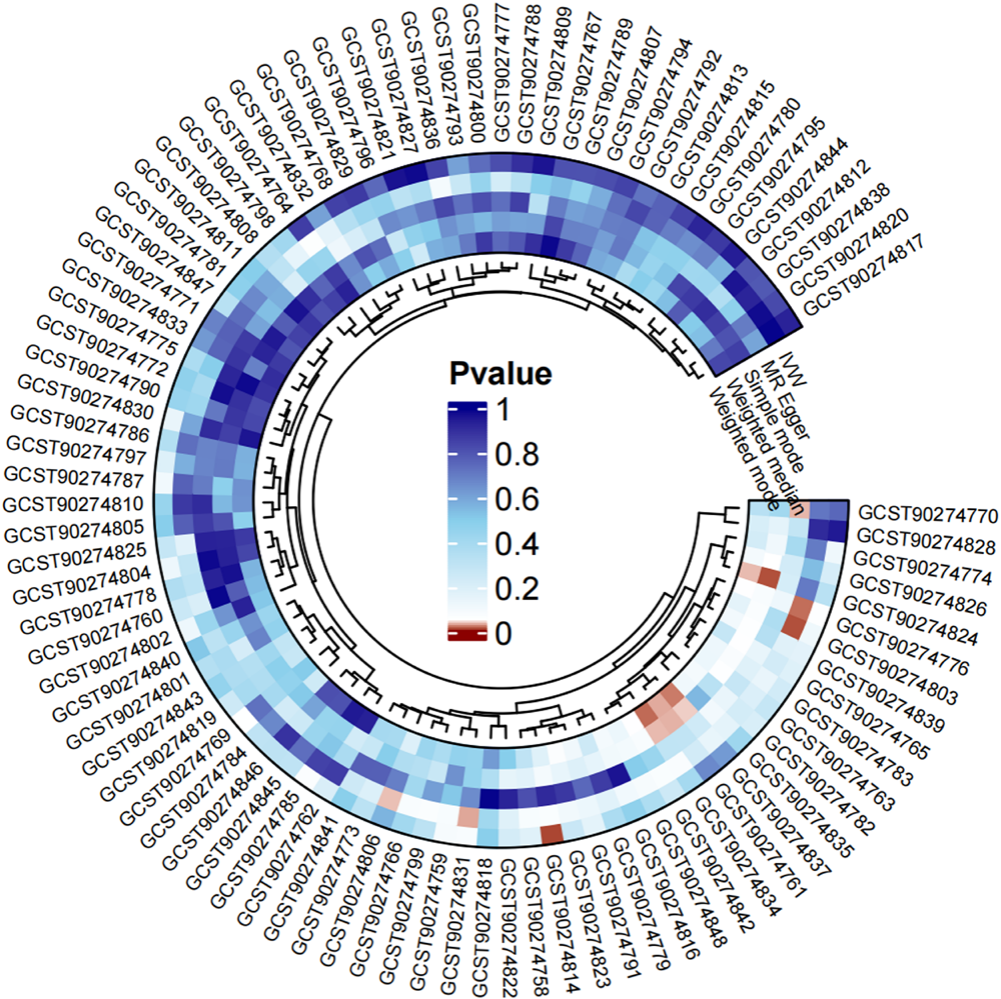
Circle diagram of causal association between SD and 91 cell-derived signaling molecules from the reverse MR analysis. MR = Mendelian randomization, SD = sleep disorders.

**Figure 5.**
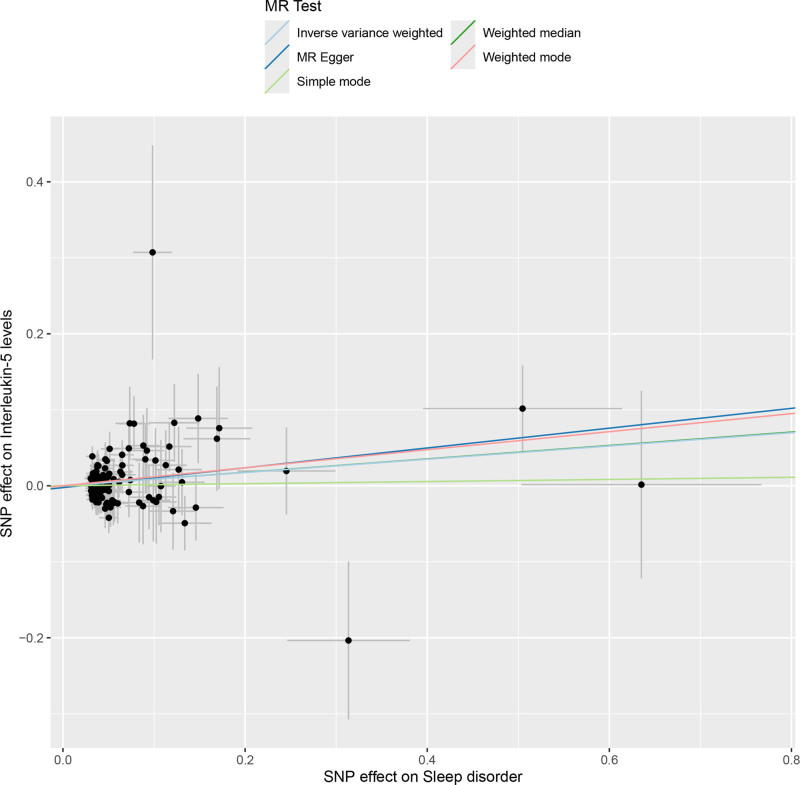
Scatter plot of the causal effect between SD and 1 positively associated cell-derived signaling molecule from the reverse MR analysis. MR = Mendelian randomization, SD = sleep disorders.

All selected SNPs involved in reverse MR analysis were robust, with *F*-statistics ranging from 19.54 to 114.76 (Table S2, Supplemental Digital Content, https://links.lww.com/MD/P869). The MR results obtained from different methods are displayed in Table S5, Supplemental Digital Content, https://links.lww.com/MD/P869. IVW outcomes indicated that SD was significantly associated with 1 cell-derived signaling molecule, while the other 90 showed the contrast (Fig. 4). To detail, interleukin-5 levels (OR = 1.091, 95% CI: 1.021–1.167, *P* = .011) were considered positively associated with SD (Fig. 5). This outcome was not detected with heterogeneity via Cochran Q test, nor any horizontal pleiotropy via MR-Egger intercept test (Table S9, Supplemental Digital Content, https://links.lww.com/MD/P869). Moreover, the LOO test proved that the MR results were not affected by any individual SNP. In summary, sensitivity analysis ensure the robustness of our outcomes.

### 3.2. Two-step MR analysis

#### 3.2.1. Effect of cell-derived signaling molecules on metabolites

All selected metabolites-related SNPs were robust instruments, with *F*-statistics ranging from 19.50 to 2297.79 (Table S3, Supplemental Digital Content, https://links.lww.com/MD/P869). Table S6, Supplemental Digital Content, https://links.lww.com/MD/P869 summarizes the IVW results, showing significant causal effects of 10 cell-derived signaling molecules on 42 metabolites (*P* < .05). Among them, the associations between CCL11 and deoxycholic acid glucuronide levels (*P* = .026) were considered positive, the same associations between CD40 and X-13684 levels (*P* = .027), mannose to mannitol to sorbitol ratio (*P* = .041), and N-cetylneuraminate to N-acetylglucosamine to N-acetylgalactosamine ratio (*P* = .028), while CD40 was negatively associated with eicosanedioate (C20-DC) levels (*P* = .018). CDCP1 showed positive correlations with gamma-glutamylcitrulline levels (*P* = .006) and N-acetylneuraminate to N-acetylglucosamine to N-acetylgalactosamine ratio (*P* = .022), but a negative correlation with branched chain 14:0 dicarboxylic acid levels (*P* = .030). GDNF showed positive relationship with 1-stearoyl-gpc (18:0) levels (*P* = .045), while negatively correlated with dopamine (DA) 3-o-sulfate levels (*P* = .020). IL-20RA was positively related to X-24978 levels (*P* = .016), while negatively to 4-guanidinobutanoate (*P* = .007), tauro-beta-muricholate (*P* = .029), 1-stearoyl-gpc (18:0) (*P* = .033), 1-arachidonoyl-GPE (20:4n6) (*P* = .004), 1-palmitoyl-GPE (16:0) (*P* = .041), 1-ribosyl-imidazoleacetate (*P* = .026), 1-(1-enyl-palmitoyl)-GPC (*P*-16:0) (*P* = .019), 6-oxopiperidine-2-carboxylate (*P* = .020), DA 3-o-sulfate (*P* = .001), N-lactoyl-tyrosine (*P* = .001), 3-(4-hydroxyphenyl) lactate (*P* = .018), and X-12680 (*P* = .000), and 2’-deoxyuridine to cytidine ratio (*P* = .033). Cochran Q outcomes indicated no evidence of heterogeneity. Although the MR-Egger intercept results suggested statistical significance for tauro-beta-muricholate levels (*P* = .044), further examination and correction using MR-PRESSO did not reveal any horizontal pleiotropy. MR-Egger intercept and MR-PRESSO test of the other 41 metabolites showed no statistical significance, performing no horizontal pleiotropy.

#### 3.2.2. Effect of metabolites on SD

Causal associations between 1400 metabolites and SD were analyzed, identifying significant causal associations for 91 metabolites, without horizontal pleiotropy (Table S7, Supplemental Digital Content, https://links.lww.com/MD/P869). Four metabolites among them showed positive association with SD, including 4-guanidinobutanoate (*P* = .039), DA 3-o-sulfate (*P* = .021), 6-oxopiperidine-2-carboxylate (*P* = .038), and X-24978 (*P* = .034). Fourteen metabolites were detected negatively associated with SD, including tauro-beta-muricholate (*P* = .020), 1-arachidonoyl-GPE (20:4n6) (*P* = .018), 1-palmitoyl-GPE (16:0) (*P* = .021), 1-ribosyl-imidazoleacetate (*P* = .043), eicosanedioate (C20-DC) (*P* = .047), gamma-glutamylcitrulline (*P* = .020), N-lactoyl tyrosine (*P* = .005), branched chain 14:0 dicarboxylic acid (*P* = .001), X-12680 (*P* = .037), deoxycholic acid glucuronide (*P* = .041), 1-(1-enyl-palmitoyl)-GPC (*P*- 16:0) (*P* = .021), 3-(4-hydroxyphenyl)lactate (*P* = .024), X-13684 (*P* = .006), and 1-stearoyl-gpc (18:0) (*P* = .025). Besides, the N-acetylneuraminate to N-acetylglucosamine to N-acetylgalactosamine ratio (*P* = .026) was positively associated with SD, while the mannose to mannitol to sorbitol ratio (*P* = .024) and 2’-deoxyuridine to cytidine ratio (*P* = .001) were found negatively associated.

#### 3.2.3. Mediation effect

In the mediation analysis involving 1400 metabolites, the levels of N-lactoyl-tyrosine were confirmed mediating the increased risk of SD associated with elevated IL-20RA levels, with a mediation effect of 0.009 (95% CI: 0.001–0.018; *P* = .034), accounting for 11.5% of the total effect.

#### 3.2.4. Sensitivity analysis

Multiple sensitivity analyses were conducted to assess potential sources of heterogeneity in our findings, with detailed results provided in Tables S6 to S9, Supplemental Digital Content, https://links.lww.com/MD/P869. While significant heterogeneity was detected in some of the results, implementing random-effects models to adjust for this variability did not materially alter the primary IVW effect estimates. To further evaluate robustness, we performed the MR-Egger regression intercept test for pleiotropy detection and applied the MR-PRESSO outlier correction procedure. These analyses confirmed the absence of directional pleiotropy in our significant associations and identified no influential outliers requiring exclusion. The consistency of results across these complementary sensitivity approaches strengthens the credibility and generalizability of our causal inferences.

## 4. Discussion

As per our information, this MR study is the first to leverage so far the largest GWAS data associated with cell-derived signaling molecules and metabolites, genetically elucidating the causal associations between 91 cell-derived signaling molecules and SD, as well as the mediating effect of metabolites in the relationships. The forward MR analysis revealed that CCL11, CDCP1, and IL-20RA are correlated positively to SD risk, while CD40 and GDNF level play contrary. Besides, the reverse MR analysis indicated that the genetic susceptibility to SD increases interleukin-5 level. Mediation analysis showed that genetically predicted N-lactoyl-tyrosine levels are crucial in mediating the causality between IL-20RA levels and the rised SD risk. Additionally, sensitivity analysis found no evidence of significant heterogeneity or pleiotropy, indicating the robustness of our results. Our findings provide valuable insights into the roles of cell-derived signaling molecules and metabolites in SD treatments.

Although the SD pathogenesis remains incompletely clarified, cell-derived signaling molecules are considered involved in the regulation of sleep rhythms. Specifically, cell-derived signaling molecules may damage the blood–brain barrier (BBB), leading to neurological complications.^[22]^ Our forward MR results indicate a prediction-based higher CCL11 level as a possible SD risk, and the result is not influenced by reverse causal effect and proven robust by sensitivity analysis. Eotaxins, including CCL11, CCL24, and CCL26, function in both physical and pathological processes of the central nervous system.^[22]^ As a key member of the eotaxins family, CCL11 is a crucial inflammatory chemokine synthesized by microglia, directly enhancing the pro-inflammatory pathways.^[22,23]^ Besides, a study has found that CCL11 selectively recruits eosinophils to inflammation sites. Following eosinophil activation, there is an increase in spontaneous reactive oxygen species (ROS) production, enhanced chemotaxis, and reduced apoptosis.^[24]^ Research suggests that CCL11 could penetrate BBB into the brain and induce ROS in microglia, leading to neuronic cytotoxic effects and multiple neurological diseases, for example, SD, anxiety, and depression.^[22,23]^ The aforementioned studies provided possible explanations for our results. CCL11 may enhance the generation of cerebral ROS via eosinophils, inducing neuronic cytotoxicity and amplifying inflammatory effect, eventually leading to the onset of SD.

CDCP1 is a transmembrane glycoprotein.^[25]^ Currently, CDCP1-associated studies has mostly focused on the field of oncology.^[26]^ To our knowledge, there has been no direct evidence found linking CDCP1 to SD. Our study genetically proves that the level-up of CDCP1 levels is significantly associated with increased SD risk. A clinical study has also indicated that CDCP1 may enhance the activity of TGF-β1 signal transducers,^[27]^ and another study has shown a positive correlation between TGF-β levels in normal epithelial breast tissue and SD.^[28]^ These studies highlighted the likeliness that CDCP1 may cause SD via increasing TGF-β activity, providing possible explanations and theoretical support for our results.

According to our forward MR outcomes, prediction-based higher genetic susceptibility to IL-20RA increased SD risk, largely mediated by N-lactoyl-tyrosine levels. The absence of significant heterogeneity and pleiotropy under sensitivity tests confirmed the robustness and reliability of these findings. As a member of the type II cellular receptor family, IL-20RA is located on chromosome 6q23. After binding to its ligands can form a functional heterodimeric receptor together with IL-20RB.^[29,30]^ However, based on current literature, no direct evidence has been found correlating the increase of IL-20RA and N-lactoyl-tyrosine levels with rising SD risk. Thus, further large-scale clinical or pre-clinical trials become necessary to deepen the investigation of causal association between IL-20RA and SD, as well as the mediating role of N-lactoyl-tyrosine levels, thereby revealing the crucial pathogenesis of SD.

CD40 is a transmembrane receptor of the tumor necrosis factor gene superfamily, and its activation can lead to the generation of cytokines, including TNF and IL-1b.^[31]^ A study has provided that CD40–CD40L signaling pathway functions in the CNS mainly by promoting inflammatory and immune responses to influence BBB permeability, leading to possible edema, microthrombus genesis, and CNS injury.^[32]^ This suggests that CD40 may influence sleep via inflammatory–response mechanisms. However, this study does not support this conclusion. Our MR outcomes indicates that CD40 was negatively correlated with SD. For the contradiction between the mentioned observational studies and our results, a possible explanation could be that observational studies are likely influenced by reverse causal effects and confounding factors, including geographical diversities, uneven age distribution, and climate varieties. In contrast, our MR study approaches the issue from a genetic perspective, which generally avoids the bias induced by these confounding factors and reverse causality, ensuring robustness and reliability. Therefore, it is necessary to perform larger clinical and pre-clinical trials to further explore the significance of CD40 in treating SD to provide more effective, evidence-based clinical guidance.

Li Wang et al found that GDNF may reduce SD risk via logistic regression analysis,^[33]^ which was confirmed in our MR analysis from genetic perspective. Mechanically, GDNF can enhance the differentiation and survival of DA neurons and regulate the generation and release of neurotransmitters by DA neurons and 5-hydroxytryptamine (5HT) neurons.^[34]^ In certain cases, 5HT may pursue awakening and inhibit rapid-eye-movement (REM) sleep, while in others, it may enhance sleeping.^[35]^ A study showed that the activation of DRN5HT neurons may relieve anxiety, thereby leading to non-REM (NREM) sleep under mild stress.^[36]^

Our reverse MR analysis confirmed that SD risk is positively associated with IL-5 levels. As a crucial cytokine in the immune responses, IL-5 participates in the regulation of the growth, differentiation, activation, and survival of eosinophils. A study has indicated that SD may be closely related to elated IL-5 levels in the body.^[37]^ Our reverse MR outcomes are in accordance with these results, furthermore providing genetic evidence to reveal the correlation between SD and rising IL-5 levels. This finding provides not only a novel perspective on SD pathogenesis but also new insights for developing SD-targeted treatments.

Our study owns several advantages. As the first genetic-based MR study to reveal the causal associations between 91 cell-derived signaling molecules and SD, our study confirms that CCL11, CDCP1, and IL-20RA are significantly associated with an increased risk of SD, while CD40 and GDNF have the opposite effect. This study is also the first involving the largest GWAS data for 2-step MR to investigate the potential mediating effect that 1400 blood metabolites have between 91 cell-derived signaling molecules and SD. As a result, it ensures that the metabolite N-lactoyl-tyrosine levels act as a latent crucial mediator in the relationships between IL-20RA and increased SD risk. Compared to clinical observational studies, our MR analysis minimizes the impact caused by reverse causal effects and confounding factors between exposures and outcomes. Besides, accompanied by sensitivity analyses, our MR analysis are reassured as robust and reliable. We selected significant genome-wide SNPs from GWAS, providing abundant and rigorously tested samples.

Limits remain, despite the rigorous execution of our current MR analysis. Firstly, to minimize the likelihood of spurious causal relationships caused by genetic-diverse individuals, the IVs involved in our study were all selected from European population and genome control studies. Thus, our results may not be applicable to other populations. Secondly, in the cascade that leads to SD, the participation of cell-derived signaling molecules is generally complex and involves latent interactions. However, MR analysis can separate their individual influence and assess the relationships between SD and these cytokines genetically. Thirdly, there are various types of SD, but the original GWAS data does not include analysis in this regard, so we are unable to conduct such analysis. Finally, although MR-PRESSO and MR-Egger intercept tests were performed to avoid confounding effects induced by pleiotropy, the residual deviation is relatively inevitable.

To conclude, our study analyzed the causal relationship between 91 cell-derived signaling molecules and SD through MR, as well as the potential mediating role of 1400 metabolites, revealing a causal relationship between 5 circulating inflammatory factors and the risk of 91 metabolites and SD.Our findings provide a new theoretical basis for understanding causal the associations between cell-derived signaling molecules and SD, discovering a new clue for developing novel therapeutic strategies targeting this certain pathological mechanism. In the future, we will continue to further investigate the specified mechanism of these metabolites, hopefully to provide more effective clinical treatment options for SD.

## 5. Conclusion

Our MR analysis elucidates the causal associations between 91 cell-derived signaling molecules and SD, proving that CCL11, CDCP1, and IL-20RA may increase SD risk, while CD40 and GDNF may decrease SD risk. Besides, N-lactoyl-tyrosine levels, the metabolite, is identified as a potential curial mediator of the relationship between IL-20RA levels and the increased SD risk. The results of this study may help better understand the pathogenesis of SD, enlightening its further treatment and prevention.

## Acknowledgments

We are thankful for the genome-wide association studies Database.

## Author contributions

**Formal analysis:** Jing Yan.

**Funding acquisition:** Sheng Xie.

**Methodology:** Chunmei Wang.

**Software:** Liqun Li, Xiaomei Tang, Jinjing Tan, Jing Yan, Jiaqi Li, Zhiwen Shen.

**Writing – original draft:** Liqun Li, Xiaomei Tang, Jinjing Tan.

## Supplementary Material


